# A designed antimicrobial peptide with potential ability against methicillin resistant *Staphylococcus aureus*

**DOI:** 10.3389/fmicb.2022.1029366

**Published:** 2022-10-10

**Authors:** Bingqian Yuan, Xiaoyu Lu, Min Yang, Qiyi He, Zhuocen Cha, Yaqun Fang, Yan Yang, Lei Xu, Jingting Yan, Ren Lai, Aili Wang, Xiaodong Yu, Zilei Duan

**Affiliations:** ^1^School of Life Sciences, Tianjin University, Tianjin, China; ^2^Key Laboratory of Animal Models and Human Disease Mechanisms of the Chinese Academy of Sciences/Key Laboratory of Bioactive Peptides of Yunnan Province, KIZ-CUHK Joint Laboratory of Bioresources and Molecular Research in Common Diseases, National Resource Center for Non-Human Primates, Kunming Primate Research Center, National Research Facility for Phenotypic & Genetic Analysis of Model Animals (Primate Facility), Sino-African Joint Research Center, and Engineering Laboratory of Peptides, Kunming Institute of Zoology, Kunming, China; ^3^The Cancer Hospital of the University of Chinese Academy of Sciences, Institute of Basic Medicine and Cancer (IBMC), Chinese Academy of Sciences, Hangzhou, Zhejiang, China; ^4^Kunming College of Life Science, University of Chinese Academy of Sciences, Beijing, China; ^5^College of Life Science, Chongqing Normal University, Chongqing, China; ^6^Department of Breast Surgery, The Third Affiliated Hospital of Kunming Medical University & Yunnan Cancer Hospital, Kunming, Yunnan, China; ^7^Yunnan Provincial Academy of Science and Technology, Kunming, China; ^8^Center for Evolution and Conservation Biology, Southern Marine Science and Engineering Guangdong Laboratory, Guangzhou, China

**Keywords:** *Staphylococcus aureus*, cathelicidin, antimicrobial peptides, GW18, drug-resistant

## Abstract

*Staphylococcus aureus* (*S. aureus*) is a Gram-positive pathogenic bacterium, which persistently colonizes the anterior nares of approximately 20–30% of the healthy adult population, and up to 60% is intermittently colonized. With the misuse and overuse of antibiotics, large-scale drug-resistant bacteria, including methicillin-resistant *S. aureus* (MRSA), have been appeared. MRSA is among the most prevalent pathogens causing community-associated infections. Once out of control, the number of deaths caused by antimicrobial resistance may exceed 10 million annually by 2050. Antimicrobial peptides (AMPs) are regarded as the best solution, for they are not easy to develop drug resistance. Based on our previous research, here we designed a new antimicrobial peptide named GW18, which showed excellent antimicrobial activity against *S. aureus*, even MRSA, with the hemolysis less than 5%, no cytotoxicity, and no acute toxicity. Notably, administration of GW18 significantly decreased *S. aureus* infection in mouse model. These findings identify GW18 as the ideal candidate against *S. aureus* infection.

## Introduction

*Staphylococcus aureus* (*S. aureus*), an opportunistic pathogen, has caused a wide range of severe clinical infections all over the world every year, which resulted in a huge burden to the health care system and seriously threatened the human health (Monaco et al., 2017). Infection of *S. aureus* may involve any part and organ of the body (Turner et al., 2019), which is the main causes of skin and soft tissue infections (Vella et al., 2021), bacteremia (Kwiecinski and Horswill, 2020), infective endocarditis (Wang et al., 2018), bone joint infections(Bouiller et al., 2020), and pleural lung related infections(Kanellakis et al., 2022).

First introduction of penicillin and methicillin show the good therapeutic effects on the infection of *S. aureus*, while the massive introduction of these antibiotics quickly leads to the emergence of antibiotics resistant strains (Chambers and Deleo, 2009). Despite the advent of vancomycin alleviating this situation (Rybak et al., 2020), vancomycin-resistant strains have been also identified with the massive use of vancomycin, and major vancomycin toxicities have been reported in the literature - in particular, nephrotoxicity and ototoxicity (Marsot et al., 2012). The widespread threat of antibiotics resistant strains forced researchers to identify and design new antimicrobial drugs.

Antimicrobial peptides (AMPs), kinds of polypeptides with antibacterial activity produced by organisms to resist the invasion of external pathogens (Mwangi et al., 2019a), are considered as the most promising choices for next-generation antibiotics due to their excellent antimicrobial activities and low tendency to induce resistance. Although many antimicrobial peptides have been identified and entered clinical trials, so far, few antimicrobial peptides have been approved by the US Food and Drug Administration (FDA) due to toxicity, stability, short half-life and high price (Chen and Lu, 2020).

Our previous studies have identified an antimicrobial peptide cathelicidin-BF from snake venom of *Bungarus fasciatus*, which exhibits potent, broad spectrum, salt-independent antimicrobial activities (Wang et al., 2008). After 10 years of efforts, cathelicidin-BF has been authorized to start clinical trials in 2018 (approval number CXHL1700235 from the Chinese National Medical Products Administration) for the treatment of colpitis caused by bacteria infection. Furthermore, a panel of synthetic AMPs based on cathelicidin-BF have been designed, which show potent antimicrobial activities with greater selectivity and security (Jin et al., 2016; Fang et al., 2019; Mwangi et al., 2019b). On this basis, here we designed a new antimicrobial peptide named GW18. Through the antibacterial activity screening, GW18 exhibited excellent antimicrobial activity against *S. aureus*, even MRSA, with little hemolysis and cytotoxicity.

## Materials and methods

### Bioinformatics analysis

Physical and chemical parameters of the GW18 was analyzed through ExPASy Bioinformatics Resource Portal.^1^ The helix-wheel structures of the peptides were constructed by HeliQuest.^2^ The multiple sequence alignment analysis of these antimicrobial peptides were analyzed using DNAMAN software.

### Peptide synthesis

GW18 (GWGAKRWGKRGWKWKRHW) and GK18 (GKRGWKRFKGAKWKRTWH) used in this experiment were synthesized by GL Biochem (Shanghai, China) with a purity of more than 95%, which was confirmed by reversed phase high-performance liquid chromatography (RP-HPLC) and mass spectrometry.

### Mice

Male C57BL/6 mice aged 6–8 weeks were purchased from Skbex Biotechnology Co. Ltd. (Henan, China). The study and all the animal experiments were approved by the Institutional Review Board and Animal Care and Use Committee at Kunming Institute of Zoology (IACUC-RE-2022-08-007).

### Bacteria strains preparation and growth conditions

The standard strain of *Staphylococcus aureus* (*S. aureus*, ATCC6538), *Escherichia coli* (*E. coli*, ATCC25922), *Pseudomonas aeruginosa* (*P. aeruginosa*, ATCC9027), *Acinetobacter baumannii* (*A. baumannii*, ATCC19606) and *Candida albicans* (*C. albicans*, ATCC/0331) used in this experiment was purchased from the microbial strain collection center of Guangdong Province. The methicillin resistant strain of *Staphylococcus aureus* (MRSA, SAZ) and clinically isolated antibiotic-resistance strains (SA115775 and SA15192) were clinically isolated from the First Affiliated Hospital of Kunming Medical University. The strains of *C. albicans* were cultured in 1/3 YM Medium (HB0297-1) + 2/3 RPMI 1640 medium (10-040-CVR, Corning), and other bacterial strains were cultured in Luria-Bertani (LB: 2 g tryptone, 1 g yeast extract, 2 g sodium chloride and 200 ml deionized water) broth.

### *In vitro* antimicrobial testing

MICs (minimal inhibitory concentration) of GW18 and clinical antibiotics (vancomycin, methicillin, colistin and fluconazol) were determined using broth dilution determination as our previous methods (Zhang et al., 2013). Briefly, bacteria strains were diluted and adjusted to the concentration of 2 × 10^5^ CFU/ml using RPMI 1640 medium. Samples were prepared as a stock solution in saline, and then diluted to a series of concentrations. 100 μl bacterial suspension and 100 μl samples at the indicated concentration were put together in 96-well plates and incubated at 37°C for 16 h. Finally, an absorbance at 600 nm was measured by a microplate reader to estimate bacterial growth. The MIC was defined as the lowest concentration where the bacterial growth could not be detected completely.

### Hemolysis and cytotoxicity assays

To evaluate possible side effects of GW18, red blood hemolysis and cytotoxicity assays were performed according to the methods as described in previous report with minor modifications (Wei et al., 2020). For hemolysis assay, human mature red blood cells were repeatedly washed with saline for 3 times, and then resuspended in saline. 100 μl human red blood cell suspension was incubated with 100 μl of GW18 at different concentrations ranging from 0 to 169.01 μM. After incubation for 30 min at 37°C, the cells were centrifuged (3,500 rpm, 5 min) and the absorbance of the supernatant was measured at 540 nm. The value for “zero hemolysis” was determined using sterile saline (negative control), while 100% hemolysis was established using 1% (v/v) Triton X-100 (positive control). Hemolysis of testing sample was calculated as the percentage of Triton X-100-induced hemolysis.

Cytotoxicity was determined using HEK293T cells from Cell Bank of Kunming Institute of Zoology, Chinese Academy of Sciences. Briefly, HEK293T cells (1 × 10^5^ cells) were plated into 96 well plates and cultured with Dulbecco’s modified Eagle’s medium (DMEM, Gibco) containing 10% Fetal Bovine Serum (FBS) and penicillin (100 U/ml)-streptomycin (100 μg/ml) at 37°C in a humidified 5% CO_2_ atmosphere. After 24 h incubation, fresh medium with GW18 at different concentration (1.32–169.01 μM) was added to the wells and incubated for another 24 h under same conditions. Cell viability was determined by adding 10 μl CCK8 in to the cell and incubated for 2 h. The absorbance at 450 nm of the solution was measured with a microplate reader. The experiments were conducted in triplicate, and 10% DMSO was considered as the positive control.

### *In vivo* acute toxicity assay

Male C57BL/6 mice aged 6–8 weeks were randomly divided into 2 groups with 10 mice in each group. GW18 (20 mg/kg and 40 mg/kg) were injected into the tail vein (intravenous injection), respectively. Then the death of mice was recorded at 12 h, 24 h, 36 h, 48 h, 60 h and 72 h, respectively.

### Bacterial killing kinetic assay

The bacterial killing kinetic assay was performed according to the previous method with minor modifications (Wei et al., 2015). *S. aureus* (ATCC6538) and methicillin resistant strain of *Staphylococcus aureus* (MRSA, SAZ) were washed three times with saline and then resuspended with RPMI 1640 medium containing 10% FBS at the concentration of 2 × 10^5^ CFU/ml. GW18 (1, 5, 10 × MIC) or vancomycin (1, 5, 10 × MIC) was added to the bacterial suspension and incubated at 37°C for 0, 15, 30, 60, 120 and 240 min, respectively. Ten microliter aliquots were extracted at each time point and diluted with fresh broth for 100 times, 100 μl of the dilution was seeded on agar plates. After incubation at 37°C for 24 h, the viable colonies were determined.

### *Staphylococcus aureus* intraperitoneal infection model

*S. aureus* (ATCC6538) and methicillin resistant strain of *Staphylococcus aureus* (MRSA, SAZ) were resuspended by saline with the concentration of 1 × 10^9^ CFU/ml. Male C57BL/6 mice aged 6–8 weeks were randomly divided into six groups as below: saline treatment group, vancomycin groups (2 mg/kg and 4 mg/kg), GW18 groups (2 mg/kg, 4 mg/kg and 8 mg/kg), and were intraperitoneally injected with 200 μl *S. aureus* or MRSA-Z, respectively. 2 h later, samples were intraperitoneally injected with the concentration indicated. After 4 h of administration, mice were sacrificed, blood was collected *via* retro-orbital bleeding and lungs were harvested and homogenized in 1 ml of saline with a hand tissue grinder. 10-fold serial dilutions of the homogenates and blood were made in pyrogen-free NaCl and equal volumes were plated on LB agar plates and incubated at 37°C. CFU were counted after 24 h.

### Effects of human plasma on GW18 antibacterial activity

We determine The stability of GW18 In human plasma according To The previously reported method (Mwangi et al., 2019b). Briefly, GW18 (final concentration 4.23 mM) was mixed with 100% human plasma for 0, 0.5, 1, 2, 4, 6, 8 and 10 h at 37°C, respectively. Residual antibacterial activity for each incubation time point was evaluated by disk diffusion assay. In brief, bacteria were seeded on nutrient agar plates, 6 mm paper disks were placed on top and 10 μl aliquots of the plasma-peptide mixture was added to the paper disks. After 24 h incubation, the inhibitory zones against *S. aureus* (ATCC6538, MRSA-Z, SA15192, SA115775) were measured and recorded.

### Plasma stability assay

To test the stability of GW18 after exposure in plasma, the peptide was first diluted in human plasma to a final concentration of 4.23 μM. After incubation for 0, 2, 4, 6, 8 and 10 h at 37°C, an aliquot of 10 μl sample was taken for protein precipitation with an equal volume of 4% H_3_PO_4_, the mixture was vortex-mixed for 2 min and then centrifuged at 13000 rpm for 15 min at 4°C. 3 μl of supernatant was analyzed using reverse-phase high performance liquid chromatography (RP-HPLC), and the remaining peptide was calculated through the integrated peak area.

### Statistical analysis

Data obtained from independent experiments were presented as mean ± standard deviation (SD). For normal continuous variables, one-way analysis of variance (ANOVA) was used. All data were analyzed using GraphPad Prism 8.0 software. Differences were considered significant at *p* < 0.05.

## Results

### Peptide design and functional screening

**Figure 1:**
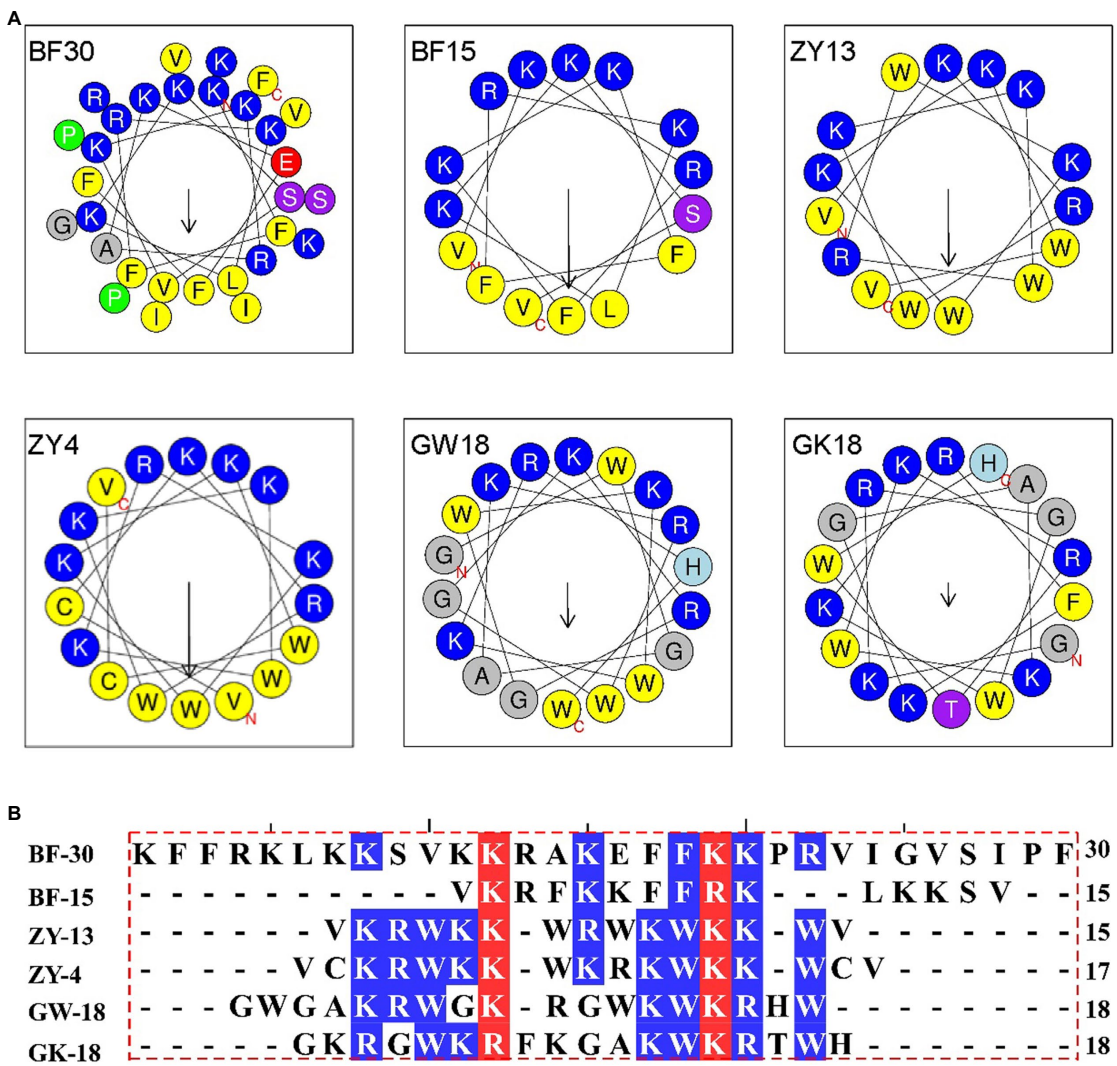
**(A)** Helical wheel projection diagrams of GW18 and other cathelicidin-BF analogs. The hydrophobic residues are presented in yellow color, positively charged hydrophilic residues blue, the noncharged polar residue purple, and negatively charged hydrophilic residue red. **(B)** The multiple sequence alignment analysis of these antimicrobial peptides were analyzed using DNAMAN software.

Cathelicidin antimicrobial peptides, which belong to the family of the host defense, exhibit a broad-spectrum effect against pathogens via direct microbicidal and immunomodulatory activities (Peng et al., 2020). On the basis of cathelicidin-BF (BF30) and its derivatives, we designed two antibacterial peptides GK18 and GW18 with the transformation strategies including: retaining the core sequence and structure of antimicrobial peptides, insertion of the hydrophobic residues and polar residues. The sequences and physicochemical properties of the GK18 and GW18 were listed in Table 1. Compared with BF-30 and its derivatives, GW18 contains the lowest net charge (+7) and GK18 contains the highest polar residues (72.22%). Similar with other antimicrobial peptides, GK18 and GW18 were also amidated at the C terminus to improve its stability. The predicted helix structures of the GK18, GW18 and other antimicrobial peptides were illustrated in Figure 1A. All the peptides could form helixes and most of them have an amphipathic structure forming a hydrophilic and hydrophobic side. Furthermore, sequence alignment of these peptides were made, and the results were illustrated in Figure 1B, which visually showed the modifications of GW18 and GW18 compared with cathelicidin-BF (BF30) and its derivatives.

**Table 1:** Physicochemical properties of the designed peptides.

Peptide	Sequence	L	NC	H	PR (n/%)	NPR (n/%)
cathelicidin-BF30	KFFRKLKKSVKKRAKEFFKKPRVIGVSIPF^a^	30	+11	0.233	16/53.33	14/46.67
cathelicidin-BF15	VKRFKKFFRKLKKSV^a^	15	+8	0.101	9/60.00	6/40.00
ZY13	VKRWKKWRWKWKKWV^a^	15	+8	0.382	8/53.33	7/46.67
ZY4	VCKRWKKWKRKWKKWCV^a^	17	+9	0.328	9/52.94	8/47.06
GW18	GWGAKRWGKRGWKWKRHW^a^	18	+7	0.261	12/66.67	6/33.33
GK18	GKRGWKRFKGAKWKRTWH^a^	18	+8	0.070	13/72.22	5/27.78

a: C-terminal amidation (-NH_2_); L: length; NC: Net charge; H: Hydrophobicity; PR: Polar residues; NPR: Nonpolar residues.

Then GK18 and GW18 were synthesized and the antimicrobial activities of them to *S. aureus* (ATCC6538), *Escherichia coli* (*E. coli*, ATCC25922), *Pseudomonas aeruginosa* (*P. aeruginosa*, ATCC9027), *Acinetobacter baumannii* (*A. baumannii*, ATCC19606) and *Candida albicans* (*C. albicans*, ATCC/0331) were tested. As shown in Supplementary Table 1, GW18 showed high activity to *S. aureus* with the minimal inhibitory concentration (MIC) value of 1.32 μM, which was lower than vancomycin with the activity against *S. aureus* with the MIC value of 1.08 μM. Besides, GW18 also exhibited antimicrobial activities against *E. coli* with the MIC values of 5.28 μM, which was lower than that of colistin and fluconazol. The antimicrobial activities of GW18 against *P. aeruginosa* and *A. baumannii* were very low with MICs higher than 20 μM, while GK18 showed no antibacterial activity against all the strains (MICs higher than 20 μM) above. From these data, we designed a specific antimicrobial peptide that inhibits *S. aureus*.

### Antimicrobial activity of GW18 against MRSA and clinically isolated antibiotic-resistance *Staphylococcus aureus* strains

To further determine the antimicrobial activity of GW18 against *S. aureus*, methicillin-resistant *S. aureus* (MRSA-Z) and two clinically isolated antibiotic-resistance strains (SA115775 and SA15192) were used in this experiment. As shown in Table 2, consistent with the activity of the standard *S. aureus* (ATCC6538), GW18 showed strong antibacterial activities against these antibiotic-resistance strains with MICs of 1.32 μM, which was similar with the activities of the clinical drug vancomycin with MICs from 0.54 μM to 2.16 μM. However, methicillin, another clinical antibiotic, only showed antimicrobial effect on standard strain *S. aureus* (ATCC6538) with a MIC of 1.94 μM, and its antimicrobial activity on the antibiotic-resistance strains was very weak with MICs from 15.53 μM to more than 124.25 μM. These data suggest GW18 exhibits potent antibacterial activity against both standard strain and antibiotic-resistance strains of *S. aureus*. Furthermore, we determined the effects of the GW18 combination with vancomycin against *S. aureus* and methicillin-resistant *S. aureus* (MRSA-Z). As illustrated in Supplementary Tables 2, 3, the combination of GW18 and vancomycin showed no synergy, while the addition of vancomycin inhibited the antimicrobial effects of GW18 against *S. aureus*.

**Table 2:** Antimicrobial activity of GW18 and clinical antibiotics against methicillin-resistant *S. aureus*.

Bacteria strains	MIC (μM)		
	GW18	Vancomycin	Methicillin
*Staphylococcus aureus* (ATCC6538)	1.32	1.08	1.94
methicillin-resistant *Staphylococcus aureus* (MRSA-Z)	1.32	0.54	>124.25
*Staphylococcus aureus* (SA115775)	1.32	1.08	62.13
*Staphylococcus aureus* (SA15192)	1.32	2.16	15.53

MIC: minimal inhibitory concentration.

### Killing kinetic of GW18 on *Staphylococcus aureus*

**Figure 2:**
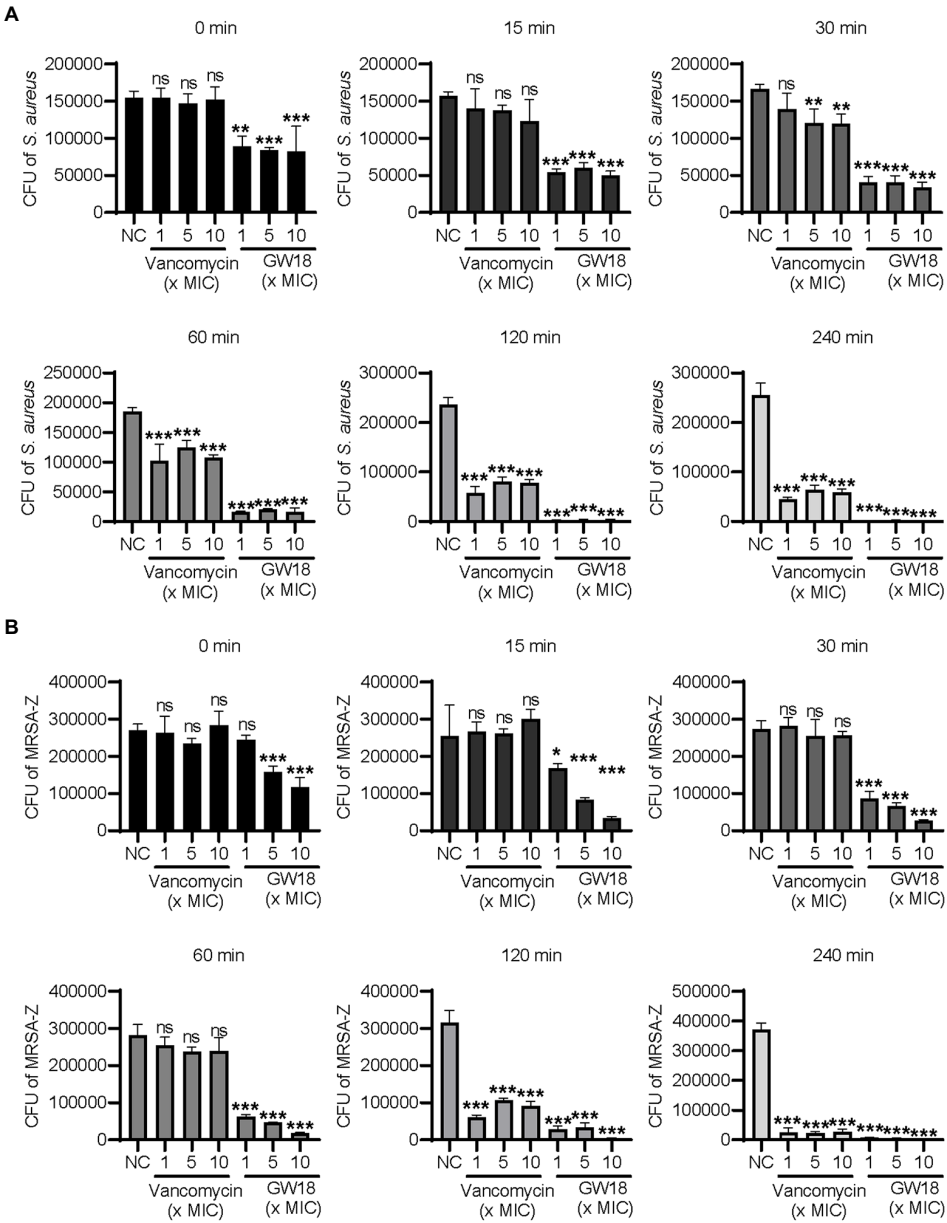
Killing kinetic of GW18 against *S. aureus*. *S. aureus* (ATCC6538, A) and methicillin-resistant *S. aureus* (MRSA-Z, B) were incubated with GW18 (1, 5, 10 × MIC) or vancomycin (1, 5, 10 × MIC) at 37°C for 0, 15, 30, 60, 120 and 240 min, respectively. Ten microliter aliquots were extracted at each time point and diluted with fresh broth for 100 times, 100 μl of the dilution was seeded on agar plates. After incubation at 37°C for 24 h, the viable colonies were determined for the killing kinetic analysis. Data represent mean ± SD values of three independent experiments. *p < 0.05, **p < 0.01, ***p < 0.001.

Bactericidal kinetics can determine the bactericidal rate of antibacterial drugs *in vitro*, and we measured the killing kinetics of GW18 on *S. aureus* (ATCC6538) and methicillin-resistant *S. aureus* (MRSA-Z). GW18 rapidly killed *S. aureus* (Figure 2A) and MRSA-Z (Figure 2B) in 1 ×, 5 ×, and 10 × MICs, which was better than that of vancomycin at the same MICs. GW18 killed almost all the *S. aureus* at 1 × MIC in 120 min, while vancomycin could not completely kill the *S. aureus* at 1 ×, 5 ×, or even 10 × MICs in 240 min. Furthermore, GW18 killed almost all the MRSA-Z at 10× MIC in 30 min, while vancomycin needed 240 min at 1 ×, 5 ×, and 10 × MICs to completely kill the MRSA-Z. Collectively, these data suggested that GW18 exhibits potent and quick antibacterial activity against *S. aureus*.

### GW18 causes a negligible hemolytic activity and cytotoxicity to mammalian cells

**Figure 3:**
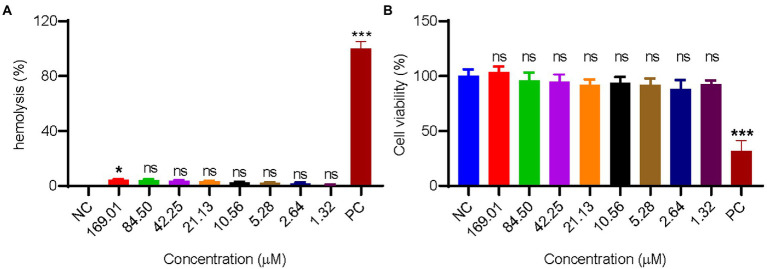
Hemolysis and cytotoxicity of GW18. **(A)** 100 μl human mature red blood cell suspension was incubated with 100 μl of GW18 at different concentrations (0–169.01 μM). After incubation for 30 min at 37°C, cells were centrifuged (3,500 rpm, 5 min) and the absorbance of the supernatant was measured at 540 nm. The value for “zero hemolysis” was determined using sterile saline (negative control), while 100% hemolysis was established using 1% (v/v) Triton X-100 (PC: positive control). Hemolysis of testing sample was calculated as the percentage of Triton X-100-induced hemolysis. **(B)** HEK293T cells (1 × 10^5^ cells) were plated into 96 well plates and incubated with GW18 (0–169.01 μM) or 10% DMSO (PC: positive control) for 24 h. Cell viability was determined by adding 10 μl CCK8 in to the cells and incubated for 2 h. The absorbance at 450 nm of the solution was measured with a microplate reader. Data represent mean ± SD of 3 independent experiments. *p < 0.05, ***p < 0.001.

Many antimicrobial peptides with excellent antibacterial activities have strong hemolytic activity, which hindered their application *in vivo*. In this experiment, the hemolytic activity of GW18 on human mature red blood cells was tested. As illustrated in Figure 3A, there is no obvious hemolytic activity of GW18 at the concentration of 1.32–169.01 μM. Besides hemolytic activity, the cytotoxicity of a drug is also an important indicator for its safety evaluation, so we investigated the cytotoxicity of GW18 on HEK293T. As shown in Figure 3B, GW18 exhibited no obvious cytotoxicity on HEK293T even at the concentration of up to 169.01 μM. Therefore, our findings suggest that the use of GW18 has a low likelihood to lead hemolytic activity and cytotoxicity of mammalian cells.

### GW18 shows no acute toxicity *in vivo*

To determine *in vivo* acute toxicity of GW18, 6–8 weeks old C57BL/6 male mice were injected with a single dose of GW18 at 20 mg/kg and 40 mg/kg, respectively, and the death of mice was recorded at 12 h, 24 h, 36 h, 48 h, 60 h and 72 h. As illustrated in Supplementary Figure 1, GW18 showed no acute toxicity, suggesting the lower toxicity of GW18. Together, these properties make GW18 an ideal candidate as a drug agent.

### GW18 maintains its antibacterial activity in plasma

**Figure 4:**
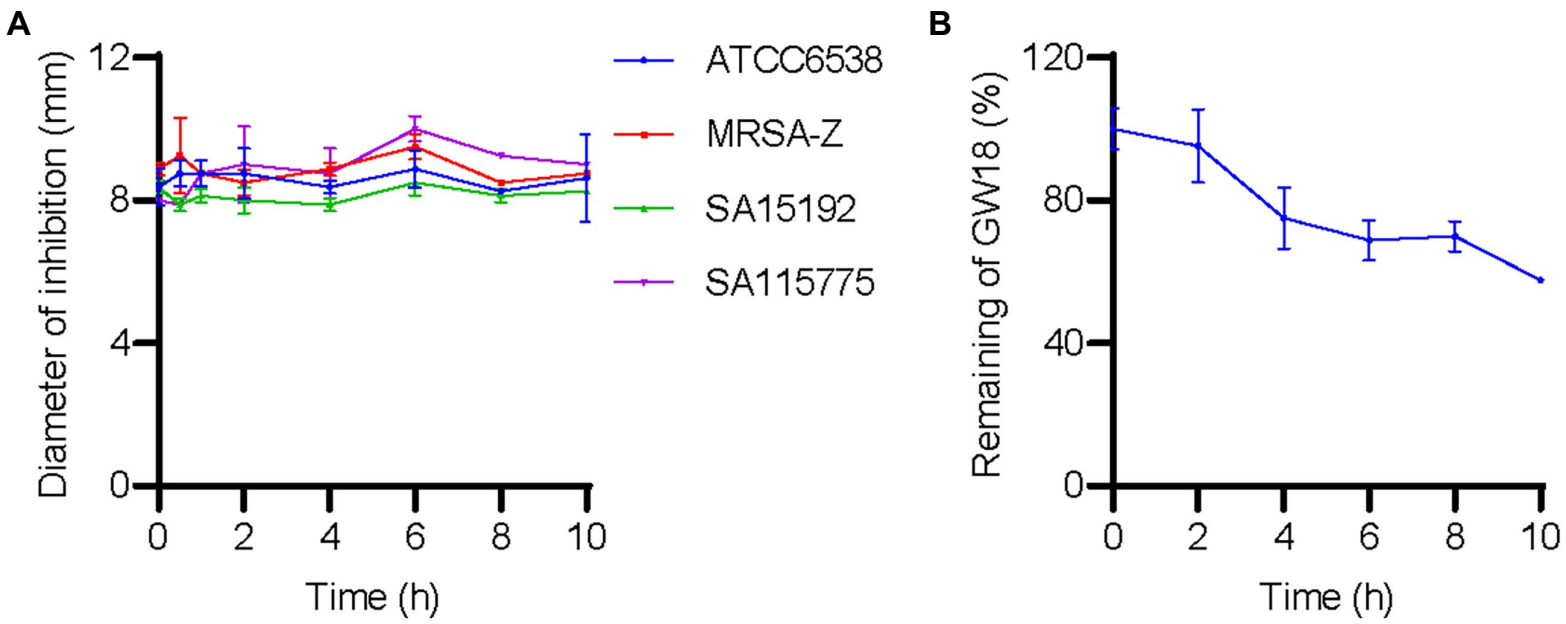
GW18 maintains its antibacterial activity against *S. aureus* in plasma. **(A)** GW18 was incubated with human plasma (final concentration 4.23 mM) and the stability of GW18 determined by evaluating the antibacterial activity against *S. aureus* (ATCC6538, MRSA-Z, SA15192, SA115775) using the disk diffusion assay by measuring the diameter of zone of inhibition after incubation for 0–10 h. **(B)** GW18 was incubated with human plasma (4.23 mM) and the remaining peptide was determined by RP-HPLC. Data represent mean ± SD of two independent experiments.

We investigated the effect of plasma on GW18’s antibacterial activity. As shown in Figure 4A, incubation of GW18 in human plasma did not significantly affect its antibacterial activity against *S. aureus*. Indeed, the inhibitory activity of GW18 against *S. aureus* standard strain (ATCC6538), clinically isolated antibiotic-resistance strains (SA115775 and SA15192), and methicillin-resistant *S. aureus* (MRSA-Z) remained even after 10 h of plasma incubation *in vitro*. Furthermore, we measured the remaining GW18 after exposure to plasma by RP-HPLC. As shown in Figure 4B, 57.63% of the actual GW18 remained after 10 h of plasma incubation *in vitro*, which suggested that some degradation of GW18 occurred. Despite the partial degradation, the antibacterial effect of GW18 against *S. aureus* did not decrease, it is possible that some degraded fragments still have the antibacterial effect, which needs to be confirmed in the future.

### GW18 suppresses the dissemination of *Staphylococcus aureus* to target organs

**Figure 5:**
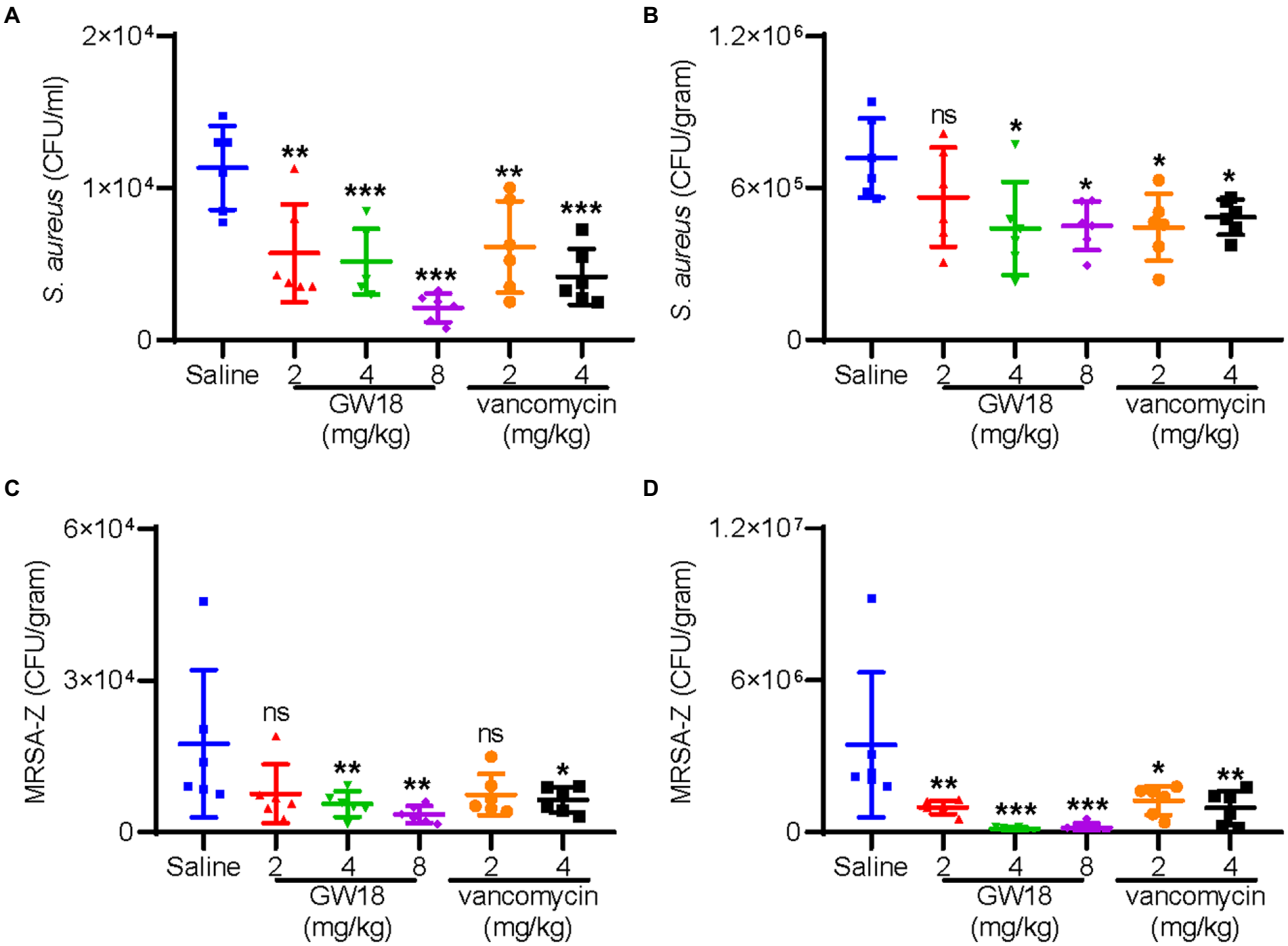
GW18 suppresses the dissemination of *S. aureus* (ATCC6538) and MRSA-Z *in vivo*. Mice (*n* = 6) were injected with *S. aureus* (ATCC6538 or MRSA-Z, 2 × 10^8^ CFU/mouse, ip), 2 h later, samples (GW18: 2, 4, 8 mg/kg; vancomycin: 2, 4 mg/kg, saline as the negative control group) were intraperitoneally injected with the concentration indicated. After 4 h of administration, mice were sacrificed, blood **(A,C)** and lung **(B,D)** were collected for the measurement of bacterial loads. Data represent mean ± SD values of six independent experiments. *p < 0.05, **p < 0.01, ***p < 0.001.

The therapeutic potential of GW18 as a candidate drug for bacteremia was evaluated. As shown in Figure 5, dissemination of standard *S. aureus* strain (ATCC6538) from the peritoneal cavity to the blood (Figure 5A) and lung (Figure 5B) was significantly suppressed with the treatment of GW18 or vancomycin. Remarkly, 2 mg/kg GW18 treatment significantly reduced the dissemination of *S. aureus* from the peritoneal cavity to the blood, which was similar with the effect of vancomycin. GW18 treatment at 8 mg/kg showed the best antimicrobial effect on *S. aureus*, which was even better than that of vancomycin at the concentration of 4 mg/kg. Furthermore, the average bacterial load in lung tissue of GW18 treatment at 2 mg/kg was reduced, and GW18 significantly suppressed the dissemination of *S. aureus* to lung at the concentration of 4 mg/kg and 8 mg/kg.

Furthermore, we also evaluated the therapeutic potential of GW18 against methicillin-resistant *S. aureus* (MRSA-Z) as a candidate drug for bacteremia. Similar with the effects on the standard *S. aureus*, GW18 significantly suppressed the dissemination of MRSA-Z from the peritoneal cavity to the blood (Figure 5C) and the lung (Figure 5D). Besides, GW18 showed higher effectivity against MRSA-Z than vancomycin at the same concentration. Taken collectively, these data confirmed the roles of GW18 in suppressing the dissemination of *S. aureus* to target organs *in vivo*.

## Discussion

Various infectious diseases caused by *S. aureus* and MRSA pose a great threat to human health (Li et al., 2022). As an important pathogenic bacteria in communities and hospitals, MRSA has shown a pandemic trend in the world (Fu et al., 2021). It is urgent to develop new antimicrobial drugs that are not prone to produce drug resistance. Based on the experience of previous research, we designed a new antimicrobial peptide GW18, which selectively inhibited the infection of *S. aureus* and MRSA.

Antimicrobial peptides (AMPs) have been identified and characterized from tissues and organisms of every kingdom and phylum, ranging from prokaryotes to humans (Yeaman and Yount, 2003; Mwangi et al., 2019a). Of the available literature, defensin, cathelicidin, hepcidin are among the best-characterized peptides, which exhibit antimicrobial activities through the directly killing bacteria, or the regulation of immune system (Ho et al., 2017). LL-37, the only human member of the cathelicidin antimicrobial peptide family, is derived from human cathelicidin antimicrobial protein 18 (hCAP18) by the cleavage of proteinase 3 (Sørensen et al., 2001). Although LL-37 has good antibacterial activity *in vivo*, elevated level of LL-37 is also reported to aggravate psoriasis (Lande et al., 2007), atherosclerosis (Edfeldt et al., 2006; Zhang et al., 2015), ulcerative colitis (Duan et al., 2018), sepsis (Fukumoto et al., 2005), thrombosis (Pircher et al., 2018; Salamah et al., 2018; Duan et al., 2022), and chronic obstructive pulmonary disease (Persson et al., 2017), which limits the use of LL-37 in these related diseases to treat bacterial infection.

In addition to endogenous LL-37, researchers have also identified many antimicrobial peptides belonging to cathelicidin family from other organisms, which provide the material basis for the design and development of new antibacterial drugs (Yu et al., 2015; Zhang et al., 2019; Zhong et al., 2020; Chai et al., 2021; Wu et al., 2021). In recent years, the designing of antimicrobial peptides based on the relationship between the structure and function of antimicrobial peptides have been confirmed the promising way to obtain novel antimicrobial peptides rapidly (Meng and Kumar, 2007; Fjell et al., 2011; Carmona et al., 2013). According to the known AMPs, the hydrophobic tryptophan (Trp) residue prefers the interfacial region of lipid bilayers, and positively changed residues, like lysine (Lys) and arginine (Arg), facilitate the interaction of the AMPs with the anionic components of the bacterial membrane (Jin et al., 2016). Furthermore, glycine (Gly) is frequently found in many antimicrobial peptides, which determines the antimicrobial activities (Van Kan et al., 2001; De Jesus Oliveira et al., 2019).

In the present study, a previously reported antimicrobial peptide cathelicidin-BF and its derivative were used as the templates to design the new antimicrobial peptide GW18, which retained the basic amino acids and replaced other residues with tryptophan or glycine. Through the functional screening, GW18 was found specifically inhibit *S. aureus* and MRSA (Table 2; Supplementary Table 1). Killing kinetics assay suggested the GW18 killed *S. aureus* even faster than vancomycin (Figure 2).

Besides the antimicrobial activity, plasma stability, hemolysis and cytotoxicity also greatly hamper the application of antimicrobial peptides (Greco et al., 2020). Negligible hemolytic activity and cytotoxicity of GW18 were detected (Figure 3). GW18 still maintained its antibacterial activity against *S. aureus* and MRSA even after incubation for 10 h in plasma (Figure 4A). Furthermore, GW18 showed no acute toxicity even at the high concentration of 40 mg/kg (Supplementary Figure 1), while it significantly suppressed the dissemination of *S. aureus* (the standard strain and MRSA) to blood and lung at the concentration of 4 mg/kg (Figure 5).

Taken collectively, the designed GW18 showed excellent antimicrobial abilities against *S. aureus* and MRSA with negligible hemolytic activity, cytotoxicity and no acute toxicity. The present study provided an excellent candidate or template for the development of therapeutic agent to treat *S. aureus* and MRSA infection.

## Data availability statement

The raw data supporting the conclusions of this article will be made available by the authors, without undue reservation.

## Ethics statement

The animal study was reviewed and approved by the Institutional Review Board and Animal Care and Use Committee at Kunming Institute of Zoology (IACUC-RE-2022-08-007).

## Author contributions

BY, XL, MY, QH, ZC, YF, YY, LX and JY conducted the experiments. RL and ZD designed the experiments, provided the guidance for the research. BY and ZD analyzed the data. BY, RL, AW, XY and ZD wrote and revised the manuscript. All authors contributed to the article and approved the submitted version.

## Funding

This work was supported by National Natural Science Foundation of China (31930015, 81770464), Chinese Academy of Sciences (SAJC202103, XDB31000000, KFJ-BRP-008), Yunnan Provincial Science and Technology Department (202002AA100007), Bureau of Industry and Information Technology of Kunming (2120784001315 and 2120784001310), and Kunming Science and Technology Bureau (2023SCP001) to RL, National Natural Science Foundation of China (U2002219) to ZD, National Natural Science Foundation of China (32002311) to YF, Yunnan Provincial Science and Technology Department (2019FB127) to ZD, Yunnan Provincial Science and Technology Department (202001AT070128) to YF, Chongqing Municipal Education Commission (HZ2021020) to XY.

## Conflict of interest

The authors declare that the research was conducted in the absence of any commercial or financial relationships that could be construed as a potential conflict of interest.

## Publisher’s note

All claims expressed in this article are solely those of the authors and do not necessarily represent those of their affiliated organizations, or those of the publisher, the editors and the reviewers. Any product that may be evaluated in this article, or claim that may be made by its manufacturer, is not guaranteed or endorsed by the publisher.

## Footnotes

^1^ http://www.expasy.org/tools/

^2^ https://heliquest.ipmc.cnrs.fr/

## References

[ref1] Bouiller K. Hocquet D. Sauget M. Bertrand X. Chirouze C. (2020). Epidemiology and risk factors of Staphylococcus aureus CC398 bone and joint infections. BMC Infect. Dis. 20:384. doi: 10.1186/s12879-020-05098-0, PMID: 32471442 PMC7260739

[ref2] Carmona G. Rodriguez A. Juarez D. Corzo G. Villegas E. (2013). Improved protease stability of the antimicrobial peptide Pin2 substituted with D-amino acids. Protein J. 32, 456–466. doi: 10.1007/s10930-013-9505-2, PMID: 23925670

[ref3] Chai J. Chen X. Ye T. Zeng B. Zeng Q. Wu J.. (2021). Characterization and functional analysis of cathelicidin-MH, a novel frog-derived peptide with anti-septicemic properties. elife 10:4411. doi: 10.7554/eLife.64411, PMID: 33875135 PMC8057816

[ref4] Chambers H. F. Deleo F. R. (2009). Waves of resistance: Staphylococcus aureus in the antibiotic era. Nat. Rev. Microbiol. 7, 629–641. doi: 10.1038/nrmicro2200, PMID: 19680247 PMC2871281

[ref5] Chen C. H. Lu T. K. (2020). Development and challenges of antimicrobial peptides for therapeutic applications. Antibiotics (Basel) 9:24. doi: 10.3390/antibiotics9010024 PMC7168295 31941022

[ref6] De Jesus Oliveira T. Oliveira U. C. Da Silva Junior P. I. (2019). Serrulin: a glycine-rich bioactive peptide from the hemolymph of the yellow Tityus serrulatus scorpion. Toxins (Basel) 11:517. doi: 10.3390/toxins11090517, PMID: 31489876 PMC6784228

[ref7] Duan Z. F. Y. Sun Y. Luan N. Chen X. Chen M. Han Y.. (2018). Antimicrobial peptide LL-37 forms complex with bacterial DNA to facilitate blood translocation of bacterial DNA and aggravate ulcerative colitis. Sci Bulletin 63, 1364–1375. doi: 10.1016/j.scib.2018.09.014 36658908

[ref8] Duan Z. Zhang J. Chen X. Liu M. Zhao H. Jin L.. (2022). Role of LL-37 in thrombotic complications in patients with COVID-19. Cell. Mol. Life Sci. 79:309. doi: 10.1007/s00018-022-04309-y, PMID: 35596804 PMC9123294

[ref9] Edfeldt K. Agerberth B. Rottenberg M. E. Gudmundsson G. H. Wang X. B. Mandal K.. (2006). Involvement of the antimicrobial peptide LL-37 in human atherosclerosis. Arterioscler. Thromb. Vasc. Biol. 26, 1551–1557. doi: 10.1161/01.ATV.0000223901.08459.57, PMID: 16645154

[ref10] Fang Y. He X. Zhang P. Shen C. Mwangi J. Xu C.. (2019). In vitro and in vivo antimalarial activity of LZ1, a peptide derived from Snake cathelicidin. Toxins (Basel) 11:379. doi: 10.3390/toxins11070379 PMC6669622 31262018

[ref11] Fjell C. D. Hiss J. A. Hancock R. E. Schneider G. (2011). Designing antimicrobial peptides: form follows function. Nat. Rev. Drug Discov. 11, 37–51. doi: 10.1038/nrd3591, PMID: 22173434

[ref12] Fu P. Xu H. Jing C. Deng J. Wang H. Hua C.. (2021). Bacterial epidemiology and antimicrobial resistance profiles in children reported by the ISPED program in China, 2016 to 2020. Microbiol Spectr 9:e0028321. doi: 10.1128/Spectrum.00283-21, PMID: 34730410 PMC8567242

[ref13] Fukumoto K. Nagaoka I. Yamataka A. Kobayashi H. Yanai T. Kato Y.. (2005). Effect of antibacterial cathelicidin peptide CAP18/LL-37 on sepsis in neonatal rats. Pediatr. Surg. Int. 21, 20–24. doi: 10.1007/s00383-004-1256-x, PMID: 15645239

[ref14] Greco I. Molchanova N. Holmedal E. Jenssen H. Hummel B. D. Watts J. L.. (2020). Correlation between hemolytic activity, cytotoxicity and systemic in vivo toxicity of synthetic antimicrobial peptides. Sci. Rep. 10:13206. doi: 10.1038/s41598-020-69995-9, PMID: 32764602 PMC7414031

[ref15] Ho J. Zhang L. Liu X. Wong S. H. Wang M. H. T. Lau B. W. M.. (2017). Pathological role and diagnostic value of endogenous host defense peptides in adult and neonatal sepsis: a systematic review. Shock 47, 673–679. doi: 10.1097/SHK.0000000000000815, PMID: 27941592

[ref16] Jin L. Bai X. Luan N. Yao H. Zhang Z. Liu W.. (2016). A designed tryptophan- and lysine/arginine-rich antimicrobial peptide with therapeutic potential for clinical antibiotic-resistant Candida albicans vaginitis. J. Med. Chem. 59, 1791–1799. doi: 10.1021/acs.jmedchem.5b01264, PMID: 26881456

[ref17] Kanellakis N. I. Wrightson J. M. Gerry S. Ilott N. Corcoran J. P. Bedawi E. O.. (2022). The bacteriology of pleural infection (TORPIDS): an exploratory metagenomics analysis through next generation sequencing. Lancet Microbe 3, e294–e302. doi: 10.1016/S2666-5247(21)00327-X, PMID: 35544066 PMC8967721

[ref18] Kwiecinski J. M. Horswill A. R. (2020). Staphylococcus aureus bloodstream infections: pathogenesis and regulatory mechanisms. Curr. Opin. Microbiol. 53, 51–60. doi: 10.1016/j.mib.2020.02.005, PMID: 32172183 PMC7244392

[ref19] Lande R. Gregorio J. Facchinetti V. Chatterjee B. Wang Y. H. Homey B.. (2007). Plasmacytoid dendritic cells sense self-DNA coupled with antimicrobial peptide. Nature 449, 564–569. doi: 10.1038/nature06116, PMID: 17873860

[ref20] Li F. Huang K. Chang H. Liang Y. Zhao J. Yang S.. (2022). A polydopamine coated nanoscale FeS theranostic platform for the elimination of drug-resistant bacteria via photothermal-enhanced Fenton reaction. Acta Biomater. 150, 380–390. doi: 10.1016/j.actbio.2022.07.046 35917910

[ref21] Marsot A. Boulamery A. Bruguerolle B. Simon N. (2012). Vancomycin: a review of population pharmacokinetic analyses. Clin. Pharmacokinet. 51, 1–13. doi: 10.2165/11596390-000000000-00000 22149255

[ref22] Meng H. Kumar K. (2007). Antimicrobial activity and protease stability of peptides containing fluorinated amino acids. J. Am. Chem. Soc. 129, 15615–15622. doi: 10.1021/ja075373f, PMID: 18041836

[ref23] Monaco M. Pimentel De Araujo F. Cruciani M. Coccia E. M. Pantosti A. (2017). Worldwide epidemiology and antibiotic resistance of Staphylococcus aureus. Curr. Top. Microbiol. Immunol. 409, 21–56. doi: 10.1007/82_2016_3, PMID: 27025380

[ref24] Mwangi J. Hao X. Lai R. Zhang Z. Y. (2019a). Antimicrobial peptides: new hope in the war against multidrug resistance. Zool. Res. 40, 488–505. doi: 10.24272/j.issn.2095-8137.2019.062, PMID: 31592585 PMC6822926

[ref25] Mwangi J. Yin Y. Wang G. Yang M. Li Y. Zhang Z.. (2019b). The antimicrobial peptide ZY4 combats multidrug-resistant Pseudomonas aeruginosa and Acinetobacter baumannii infection. Proc. Natl. Acad. Sci. U. S. A. 116, 26516–26522. doi: 10.1073/pnas.1909585117, PMID: 31843919 PMC6936460

[ref26] Peng L. Du W. Balhuizen M. D. Haagsman H. P. De Haan C. A. M. Veldhuizen E. J. A. (2020). Antiviral activity of chicken cathelicidin B1 against influenza a virus. Front. Microbiol. 11:426. doi: 10.3389/fmicb.2020.00426, PMID: 32265870 PMC7096384

[ref27] Persson L. J. Aanerud M. Hardie J. A. Miodini Nilsen R. Bakke P. S. Eagan T. M.. (2017). Antimicrobial peptide levels are linked to airway inflammation, bacterial colonisation and exacerbations in chronic obstructive pulmonary disease. Eur. Respir. J. 49:1601328. doi: 10.1183/13993003.01328-2016, PMID: 28298400

[ref28] Pircher J. Czermak T. Ehrlich A. Eberle C. Gaitzsch E. Margraf A.. (2018). Cathelicidins prime platelets to mediate arterial thrombosis and tissue inflammation. Nat. Commun. 9:1523. doi: 10.1038/s41467-018-03925-2, PMID: 29670076 PMC5906636

[ref29] Rybak M. J. Le J. Lodise T. P. Levine D. P. Bradley J. S. Liu C.. (2020). Executive summary: therapeutic monitoring of vancomycin for serious methicillin-resistant Staphylococcus aureus infections: a revised consensus guideline and review of the American Society of Health-System Pharmacists, the Infectious Diseases Society of America, the Pediatric Infectious Diseases Society, and the Society of Infectious Diseases Pharmacists. Pharmacotherapy 40, 363–367. doi: 10.1002/phar.2376, PMID: 32227354

[ref30] Salamah M. F. Ravishankar D. Kodji X. Moraes L. A. Williams H. F. Vallance T. M.. (2018). The endogenous antimicrobial cathelicidin LL37 induces platelet activation and augments thrombus formation. Blood Adv. 2, 2973–2985. doi: 10.1182/bloodadvances.2018021758, PMID: 30413433 PMC6234361

[ref31] Sørensen O. E. Follin P. Johnsen A. H. Calafat J. Tjabringa G. S. Hiemstra P. S.. (2001). Human cathelicidin, hCAP-18, is processed to the antimicrobial peptide LL-37 by extracellular cleavage with proteinase 3. Blood 97, 3951–3959. doi: 10.1182/blood.V97.12.3951, PMID: 11389039

[ref32] Turner N. A. Sharma-Kuinkel B. K. Maskarinec S. A. Eichenberger E. M. Shah P. P. Carugati M.. (2019). Methicillin-resistant Staphylococcus aureus: an overview of basic and clinical research. Nat. Rev. Microbiol. 17, 203–218. doi: 10.1038/s41579-018-0147-4, PMID: 30737488 PMC6939889

[ref33] Van Kan E. J. Van Der Bent A. Demel R. A. De Kruijff B. (2001). Membrane activity of the peptide antibiotic clavanin and the importance of its glycine residues. Biochemistry 40, 6398–6405. doi: 10.1021/bi0028136, PMID: 11371202

[ref34] Vella V. Galgani I. Polito L. Arora A. K. Creech C. B. David M. Z.. (2021). Staphylococcus aureus skin and soft tissue infection recurrence rates in outpatients: a retrospective database study at 3 US medical centers. Clin. Infect. Dis. 73, e1045–e1053. doi: 10.1093/cid/ciaa1717, PMID: 33197926 PMC8423503

[ref35] Wang A. Gaca J. G. Chu V. H. (2018). Management considerations in infective endocarditis: a review. JAMA 320, 72–83. doi: 10.1001/jama.2018.7596, PMID: 29971402

[ref36] Wang Y. Hong J. Liu X. Yang H. Liu R. Wu J.. (2008). Snake cathelicidin from Bungarus fasciatus is a potent peptide antibiotics. PLoS One 3:e3217. doi: 10.1371/journal.pone.0003217, PMID: 18795096 PMC2528936

[ref37] Wei L. Gao J. Zhang S. Wu S. Xie Z. Ling G.. (2015). Identification and characterization of the first cathelicidin from sea snakes with potent antimicrobial and anti-inflammatory activity and special mechanism. J. Biol. Chem. 290, 16633–16652. doi: 10.1074/jbc.M115.642645, PMID: 26013823 PMC4505416

[ref38] Wei H. Xie Z. Tan X. Guo R. Song Y. Xie X.. (2020). Temporin-like peptides show antimicrobial and anti-biofilm activities against Streptococcus mutans with reduced hemolysis. Molecules 25:724. doi: 10.3390/molecules25235724, PMID: 33291521 PMC7730238

[ref39] Wu J. Zhang H. Chen X. Chai J. Hu Y. Xiong W.. (2021). FM-CATH, a novel cathelicidin from Fejervarya Multistriata, shows therapeutic potential for treatment of CLP-induced sepsis. Front. Pharmacol. 12:731056. doi: 10.3389/fphar.2021.731056, PMID: 34483941 PMC8415707

[ref40] Yeaman M. R. Yount N. Y. (2003). Mechanisms of antimicrobial peptide action and resistance. Pharmacol. Rev. 55, 27–55. doi: 10.1124/pr.55.1.2 12615953

[ref41] Yu H. Lu Y. Qiao X. Wei L. Fu T. Cai S.. (2015). Novel Cathelicidins from pigeon highlights evolutionary convergence in Avain Cathelicidins and functions in modulation of innate immunity. Sci. Rep. 5:11082. doi: 10.1038/srep11082, PMID: 26194630 PMC4508531

[ref42] Zhang L. Jie H. Xiao Y. Zhou C. Lyu W. Bai W. (2019). Genomic identification and expression analysis of the cathelicidin gene family of the Forest musk deer. Animals (Basel) 9:481. doi: 10.3390/ani9080481 PMC6719980 31344924

[ref43] Zhang Z. Meng P. Han Y. Shen C. Li B. Hakim M. A.. (2015). Mitochondrial DNA-LL-37 complex promotes atherosclerosis by escaping from Autophagic recognition. Immunity 43, 1137–1147. doi: 10.1016/j.immuni.2015.10.018, PMID: 26680206

[ref44] Zhang Z. Mu L. Tang J. Duan Z. Wang F. Wei L.. (2013). A small peptide with therapeutic potential for inflammatory acne vulgaris. PLoS One 8:e72923. doi: 10.1371/journal.pone.0072923, PMID: 24013774 PMC3755965

[ref45] Zhong L. Liu J. Teng S. Xie Z. (2020). Identification of a novel cathelicidin from the Deinagkistrodon acutus genome with antibacterial activity by multiple mechanisms. Toxins (Basel) 12:771. doi: 10.3390/toxins12120771, PMID: 33291852 PMC7762006

