# Supplementary material for: A designed antimicrobial peptide with potential ability against methicillin resistant Staphylococcus aureus
Source: Front Microbiol. 2022 Oct 10;13:1029366. doi: 10.3389/fmicb.2022.1029366 (PMC9589885; doi:10.3389/fmicb.2022.1029366)
Supplement: Supplementary file 1 [file Data_Sheet_1.docx]

Supplementary Material

# Supplementary Figures and Tables

## Supplementary Figure

**
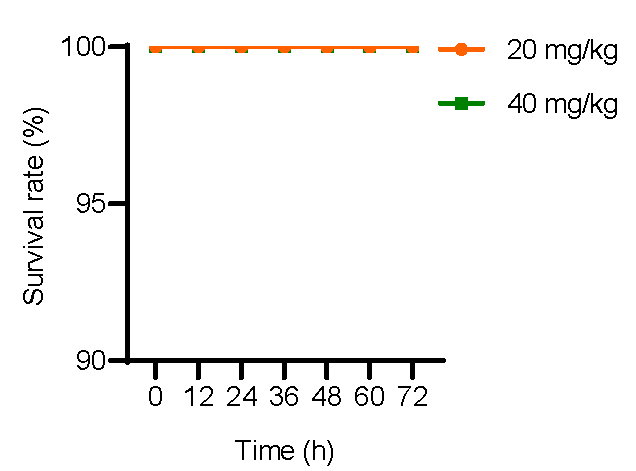
Supplementary Figure 1.** The acute toxicity of GW18. Male C57BL/6 mice aged 6–8 weeks were randomly divided into 2 groups (n = 10). GW18 (20 mg/kg and 40 mg/kg) were intravenously injected into the tail vein, respectively. Then the death of mice was recorded at 12 h, 24 h, 36 h, 48 h, 60 h and 72 h respectively.

**Supplementary Table 1.** Antimicrobial activity of GW18 and clinical antibiotics against standard bacterial strains

|  | MIC (μM) | | | | |
| --- | --- | --- | --- | --- | --- |
| Bacterial strains | GW18 | GK18 | Vancomycin | Colistin | Fluconazol |
| *Staphylococcus aureus* (ATCC6538) | 1.32 | >20 | 1.08 |  |  |
| *Escherichia coli* (ATCC25922) | 5.28 | >20 |  | 0.56 |  |
| *Pseudomonas aeruginosa* (ATCC9027) | >20 | >20 |  | 0.56 |  |
| *Acinetobacter baumannii* (ATCC19606) | >20 | >20 |  | 0.56 |  |
| *Candida albicans* (ATCC/0331) | >20 | >20 |  |  | 1.27 |

**Supplementary Table 2.** Antimicrobial activity of GW18 combination with vancomycin against standard bacterial strain of *Staphylococcus aureus* (ATCC6538).

| *S. aureus* (ATCC6538) | | GW18 (MIC) | | | |
| --- | --- | --- | --- | --- | --- |
|  |  | 1 | 0.5 | 0.25 | 0.125 |
| vancomycin (MIC) | 1 | + | + | + | + |
|  | 0.5 | - | - | - | - |
|  | 0.25 | - | - | - | - |
|  | 0.125 | - | - | - | - |

+: *S. aureus* (ATCC6538) was significantly inhibited;

-: *S. aureus* (ATCC6538) was not inhibited.

**Supplementary Table 3.** Antimicrobial activity of GW18 combination with vancomycin against methicillin-resistant *Staphylococcus aureus* (MRSA-Z).

| methicillin-resistant *Staphylococcus aureus* (MRSA-Z) | | GW18 (MIC) | | | |
| --- | --- | --- | --- | --- | --- |
|  |  | 1 | 0.5 | 0.25 | 0.125 |
| vancomycin (MIC) | 1 | + | + | + | + |
|  | 0.5 | - | - | - | - |
|  | 0.25 | - | - | - | - |
|  | 0.125 | - | - | - | - |

+: methicillin-resistant *Staphylococcus aureus* (MRSA-Z) was significantly inhibited;

-: methicillin-resistant *Staphylococcus aureus* (MRSA-Z) was not inhibited.
